# Molecular Profiling of Tissue Samples with Chronic Rejection from Patients with Chronic Lung Allograft Dysfunction: A Pilot Study in Cystic Fibrosis Patients

**DOI:** 10.3390/biom13010097

**Published:** 2023-01-03

**Authors:** Francesca Lunardi, Daniela Isabel Abbrescia, Luca Vedovelli, Federica Pezzuto, Francesco Fortarezza, Giovanni Maria Comacchio, Vincenza Guzzardo, Pia Ferrigno, Monica Loy, Chiara Giraudo, Anna Sara Fraia, Eleonora Faccioli, Fausto Braccioni, Emanuele Cozzi, Dario Gregori, Geert M. Verleden, Fiorella Calabrese, Francesco Paolo Schena, Federico Rea

**Affiliations:** 1 Department of Cardiac, Thoracic, Vascular Sciences and Public Health, University of Padova, 35128 Padova, Italy; 2 Schena Foundation, University Hospital of Bari, 70124 Bari, Italy; 3 Department of Medicine, University of Padova, 35128 Padova, Italy; 4 Department of Respiratory Diseases, University Hospitals Leuven, 3000 Leuven, Belgium

**Keywords:** lung transplantation, chronic lung allograft dysfunction, transbronchial biopsy, transcriptomics, RNA sequencing

## Abstract

Chronic rejection (CR) is the main culprit for reduced survival and quality of life in patients undergoing lung transplantation (Ltx). High-throughput approaches have been used to unveil the molecular pathways of CR, mainly in the blood and/or in bronchoalveolar lavage. We hypothesized that a distinct molecular signature characterizes the biopsies of recipients with clinically confirmed histological signs of CR. Eighteen cystic fibrosis patients were included in the study and RNA sequencing was performed in 35 scheduled transbronchial biopsies (TBBs): 5 with acute cellular rejection, 9 with CR, and 13 without any sign of post-LTx complication at the time of biopsy; 8 donor lung samples were used as controls. Three networks with 33, 26, and 36 differentially expressed genes (DEGs) were found in TBBs with CR. Among these, seven genes were common to the identified pathways and possibly linked to CR and five of them (LCN2, CCL11, CX3CL1, CXCL12, MUC4) were confirmed by real-time PCR. Immunohistochemistry was significant for LCN2 and MUC4. This study identified a typical gene expression pattern in TBBs with histological signs of CR and the LCN2 gene appeared to play a central role. Thus, it could be crucial in CR pathophysiology.

## 1. Introduction

Lung transplantation (LTx) is the only effective therapeutic option for patients with end-stage lung diseases. According to the latest report from the registry of the International Society for Heart and Lung Transplantation (ISHLT), more than 4500 lung transplants are performed yearly worldwide, with about 1400 cases in Europe [1]. Chronic lung allograft dysfunction (CLAD) represents the primary cause of the poor long-term outcome of LTx, occurring in approximately 50% of patients within five years after Ltx [2,3]. According to the latest consensus report, CLAD is defined as a heterogeneous condition comprising two major distinct phenotypes, namely bronchiolitis obliterans syndrome (BOS) and restrictive allograft syndrome (RAS) [2,4]. Several immunological and non-immunological risk factors have been associated with CLAD, including innate and adaptive immunity, both T cell-mediated and humoral. Indeed, acute cellular rejection (ACR), lymphocytic bronchiolitis, and antibody-mediated rejection (AMR) are all risk factors for the subsequent development of CLAD [5,6].

When CLAD is diagnosed, it is, per definition, an irreversible condition, and re-transplantation may be the only effective treatment option, especially in cystic fibrosis (CF) patients. Indeed, they are transplanted at a very young age and, even if they show the longest survival after LTx, they often undergo a re-transplantation when CLAD occurs. Re-transplantation is a procedure that can strongly impact patient quality of life and health costs. Thus, better knowledge of CLAD pathogenesis in these patients could result in better management, delaying the need for a re-transplantation.

Even though several attempts have been made at preventing CLAD and developing more effective therapeutic approaches, solid bases for firm recommendations and treatment guidelines are still lacking. Therefore, it remains necessary to unravel the molecular mechanisms leading to CLAD with the ultimate goal of identifying early biomarkers for diagnosis and targeted treatment. During the last decade, a few research studies have used high throughput techniques to investigate biomarker predictors for CLAD, mainly microarray or real-time polymerase chain reaction (RT-PCR) [7,8,9,10]. Although these data were instrumental in identifying the molecular pathways involved in CLAD, the results had some limitations: (i) the number of genes studied by PCR was small; (ii) those genes were involved in a limited number of cellular processes; (iii) some genes expected to be altered were not considered. Omics technologies have been widely used recently in life sciences, in order to discover transcripts for future validation with other methodological approaches and/or in a greater study population. The science of omics in transplantation is relatively novel, particularly in LTx. To the best of our knowledge, only one study performed RNA-sequencing and Nanostring digital RNA counting in airway brushing and transbronchial biopsies (TBB) from recipients with lymphocytic bronchiolitis, a lesion that is supposed to precede CLAD [11,12].

High throughput technologies, such as those used in our study (RNA-sequencing and Real-Time PCR) provide large-scale data for elucidating gene function, gene products, and cell systems. It is well known that these approaches often require very large sample sizes and the optimal one can be determined using power calculations in software tools. In case of small study populations (such as in pilot studies, in rare diseases, or in case of invasive tools) it is mandatory to well characterize the case series and the biological material, reducing biases and possible confounding factors.

We hypothesized that a distinct molecular signature occurs in TBB when there are typical histological signs of chronic rejection (CR). Thus, the main goal of the study was to advance the understanding of CLAD pathophysiology using high-throughput technologies so as to identify transcripts for future validation and introduction as early diagnostic and therapeutic markers. In particular, the study only includes patients undergoing LTx for CF in order to have a homogeneous population in terms of age, etiopathogenesis, and comorbidities.

## 2. Materials and Methods

### 2.1. Study Design and Population

**Figure 1:**
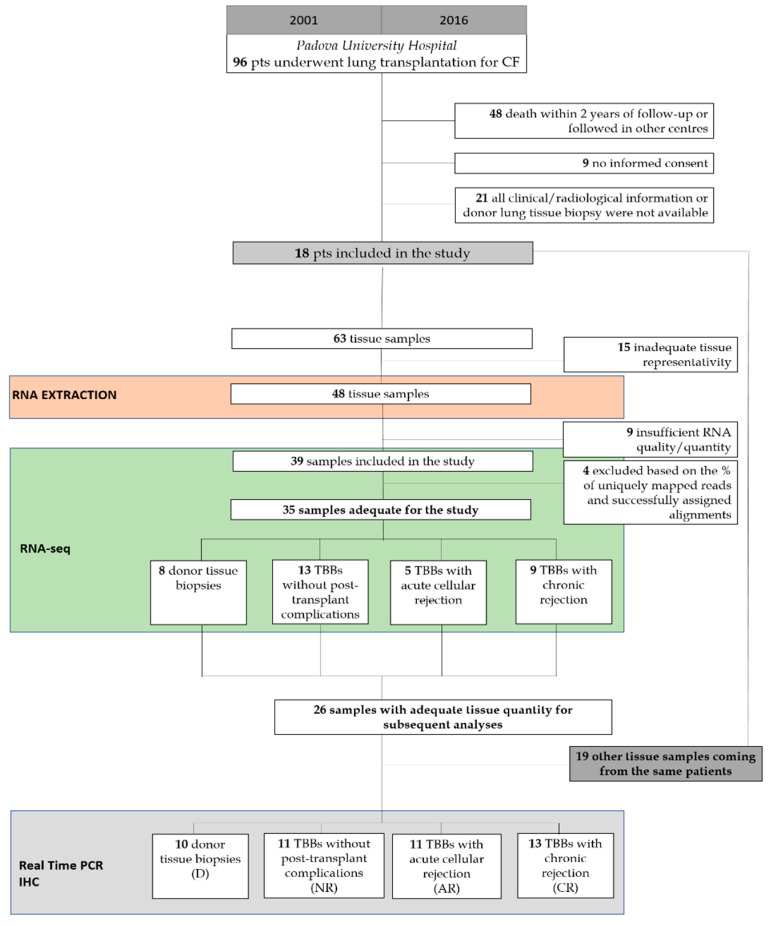
CONSORT diagram showing the study population and design.

Ninety-six patients with CF underwent LTx from 2001 to 2016 in the Thoracic Surgery Unit of the University Hospital of Padova, Italy. This observational study included 18 patients, according to the following inclusion criteria: (1) at least two years of follow-up; (2) availability of donor lung biopsy; (3) availability of all clinical-functional information; and (4) informed consent (Figure 1, Table 1). The study was designed in accordance with the Declaration of Helsinki and patients/relatives of lung donors gave informed consent for research purposes. The Institutional Ethics Committee approved the study (AOP2224, 18 February 2021).

Immunosuppression and prophylaxis treatments were given following the center’s protocols [8]. Patients were monitored with a scheduled protocol of surveillance, consisting of TBB and bronchoalveolar lavages (BALs) [13]. At each time point, clinical assessment consisted of spirometry in conjunction with blood gas analysis, measurement of immunosuppressive drug levels, chest radiographs, and/or computed tomography (CT).

### 2.2. Sample Collection

Sixty-three scheduled TBBs were collected in the follow-up of the patients and 35 of them were included in the molecular investigation (mean ± SD: 1.8 ± 0.6 per patient), mainly based on the histological features. There were 8 donor lung biopsies (D), 13 TBBs without any sign of post-transplant complication (No rejection-NR), 5 TBBs with ACR (AR), and 9 TBBs with chronic rejection signs (CR) (Figure 1). TBBs did not show any other post-transplant complication (e.g., infection, ischemia/reperfusion injury, etc.). All the biopsies with no rejection and acute rejection were taken in the first post-transplant year, excluding those of the first month. CR samples were taken within the first 4 years post-LTx.

We considered lung biopsies highly suggestive of CR when they showed features of obliterative bronchiolitis (grade C), pleuro-parenchymal fibroelastosis (PPFE), late diffuse alveolar damage/organizing pneumonia, and interstitial fibrosis with nonspecific interstitial pneumonia-like (NSIP) features.

In all cases, ancillary techniques (special stains, immunohistochemistry, and molecular analyses) routinely used in pathology labs are which are mandatory for a more sensitive and specific diagnosis of these post-transplant complications were employed. In any case, multidisciplinary discussions with other specialists (pulmonologists, immunologists, infectious disease specialists, and radiologists) were always performed for a final diagnosis.

### 2.3. RNA Extraction

Formalin-fixed paraffin-embedded (FFPE) blocks were cut into 4–8 μm thick sections and processed with standard hematoxylin-eosin staining. RNase contamination was avoided by cleaning surfaces and tools with RNaseZap™ RNase Decontamination Solution (ThermoFisher Scientific, Carlsbad, CA, USA). The total RNA was extracted immediately after microdissection using an RNeasy^®^ FFPE kit (Qiagen, Hilden, Germany) according to the manufacturer’s protocol. RNA yield and quality were determined by UV adsorption on a NanoDrop 1000 Spectrophotometer and fragment size was analyzed using the RNA 6000 Pico assay (Agilent Technologies, Santa Clara, CA, USA) run on the 2100 Bioanalyzer. RNA quality was assessed using DV200 values and only cases with DV200 ≥ 41% were included for library preparation.

### 2.4. RNA Sequencing and Differential Gene Expression Analysis

Next generation sequencing experiments, including sample quality control, were performed by the start-up Genomix4Life S.r.l. (Baronissi, Salerno, Italy). Indexed libraries were prepared from 20 ng of each purified RNA sample using the TruSeq RNA Exome Sample Prep Kit (Illumina). Libraries were quantified by the TapeStation 4200 (Agilent Technologies) and the Invitrogen Qubit fluorometer (Thermo Fisher Scientific). The pooled samples were used for cluster generation and sequencing using the Illumina NextSeq 500 System (Illumina) in a 2 × 75 paired-end format at a final concentration of 1.8 pmol. FastQ underwent quality control using the FastQC tool [14]. The Cutadapt tool (version 2.5) [15] was used to remove the adapter sequence and the very short reads (read length < 20). The mapping of paired-end reads was performed using the STAR tool (version 2.7.2b) [16], with the standard parameters for paired reads, on the reference genome assembly hg38, obtained from GenCode (version v.29) [17]. The quantification of transcripts expressed in each sample was performed using the FeatureCount algorithm tool (version 2.0) [18,19]. Multiple samples per patients were modeled in the DESeq model matrix as repeated values. The DESeq2 tool [20] was used to perform the normalization matrix and differentially expressed genes (DEGs) were evaluated in each sample. Euclidean distances (heat map distances) and principal component analysis (PCA) on all samples in each condition were considered to evaluate the general similarity between samples. After PCA, samples recognized as outliers according to Tukey’s rule (1.5 × interquartile range) were removed without compromising the number of samples necessary to preserve the significance of our statistical analysis, as reported by the ENCODE Guidelines and Best Practices for RNA-Seq [21,22]. This approach was necessary to better identify the biological pathways and networks associated with a specific histological diagnosis in the follow-up TBB.

### 2.5. Ingenuity Pathway Analysis (IPA)

IPA software (Ingenuity System, Redwood City, CA, USA: http://www.ingenuity.com, accessed on 4 February 2020) was used to assess biological relationships among genes and entities with a fold change (FC) > 1.5 and false discovery rate (FDR) < 0.05. Network analysis was done comparing different groups of samples. The overlay between the most representative canonical pathways onto the top ranked network gave the most representative gene sets. The most significant genes were sorted according to a common biological function using the Molecular Signatures Database, Broad Institute software (http://software.broadinstitute.org/gsea/, accessed on 4 February 2020). Gene Set Enrichment Analysis (GSEA), a computational method that determines a priori whether a defined set of genes belongs to a specific curated molecular pathway, was performed to assess the involvement of the selected genes in CR.

### 2.6. Quantitative Real-Time (qRT-PCR) Analysis

qRT-PCR was used to validate genes that were found significantly dysregulated at the RNA sequencing analysis (adjusted-*p* value < 0.05) in the same tissue specimens. It was performed in 45 samples to validate the selected genes: 10 D, 11 NR, 11 AR, and 13 CR.

After reverse transcription, cDNA libraries were amplified by PCR and purified of residual primers and nucleotides with the Qiaquick PCR Purification Kit (Qiagen) according to the manufacturer’s instructions. Real-time PCRs were performed using the StepOnePlus Real-Time PCR System (Thermo Fisher Scientific) and the SensiMix SYBR Hi-ROX Kit (Bioline) in triplicate. β-actin gene amplification was used as a reference standard to normalize the target signal. Amplification specificity was confirmed by a melting curve and the amount of mRNA target was evaluated using the comparative cycle threshold (ΔCt) method. All data were expressed as 2^−ΔΔCt^ and outlier values were identified through the Smirnov-Grubbs test available on R Commander (Version 2.5-1). Data are expressed as mean ± standard deviation (SD). The Mann–Whitney test was used to assess differences between two groups and the Kruskal-Wallis test (Dunn’s Multiple Comparison Test) was used to evaluate statistically significant differences between groups. The chi-squared or Fisher’s test were used for categorical variables. All statistical analyses and graphs were generated with GraphPad Prism 5.0 (GraphPad Software, San Diego, CA, USA) and the threshold for significance was set at two-tailed 0.05.

### 2.7. Immunohistochemistry and Multiplex Immunofluorescence

Validation using immunohistochemistry (IHC) was performed in 45 lung tissue samples (the same as those analyzed by Real-Time) and an additional eight explanted lung samples with CR signs. Immunohistochemistry (IHC) was performed for some of the overexpressed genes following the antibody manufacturer’s protocol. Three μm-thick sections were processed for immunohistochemical analysis using the following monoclonal antibodies: anti-LCN2 (clone: 220310, R&D Systems, Minneapolis, MN, USA), anti-CCL11 (clone: 43911, Thermo Fisher Scientific, Waltham, MA, USA), anti-CX3CL1 (clone: 81513, R&D Systems, Minneapolis, MN, USA), anti-CXCL12 (clone: 79018, R&D Systems, Minneapolis, MN, USA), and anti-MUC4 (clone: 1G8, Thermo Fisher Scientific, Waltham, MA, USA) in the Leica Bond-III Autostainer (Leica Microsystems Srl, Wetzlar, Germany) according to the manufacturer’s protocol. Finally, the sections were counterstained with Mayer‘s haematoxylin. Immunostaining was expressed as scores ranging from 0–3, evaluated in the different types of cells. In addition, a morphometric quantification of LCN2 and MUC4 was done in the explanted lung samples with CR signs by using Image Pro-Plus software and expressing positive areas as a percentage of the total tissue area.

Moreover, multiplex immunofluorescence with Opal 3-Plex Detection Kits (Akoya Biosciences, Marlborough, MA, USA) with the following primary antibodies was performed according to the manufacturer’s protocol: anti-CD68/anti-AE1AE3/anti-LCN2 (for co-localization of LCN with macrophages and epithelial cells) and anti-CD68/anti-CD31/anti-MUC4 (for co-localization of MUC4 with macrophages and endothelial cells).

## 3. Results

### 3.1. Comprehensive Profiling of Biopsies Reveals Specific Alterations

Based on RNA quality and quantity, RNA sequencing analysis was completed in 35 tissue samples (8 D, 13 NR, 5 AR, and 9 CR). PCA analysis of the RNA sequencing data was performed to comparatively characterize the overall transcriptome profiles. To this end, the PCA plots revealed distinct expression profiles corresponding to the two-by-two comparisons that were made. After the removal of the outliers 28 samples were left (8 D, 8 NR, 5 AR, and 7 CR).

Differential expression analysis showed more than 15,000 DEGs in TBBs with the different histological diagnoses compared with lung donor samples (Table 2).

For both AR and CR, unlike for D, a very large activation of kinases was in place, with PRKACG orchestrating most of the modifications. Moreover, inflammatory immune response (with IFNA4, 6, 7, 8, 13, 14, 17, 21, 16, and 10) was also heavily involved and activated, while surfactant production and secretion was probably impaired (i.e., surfactant proteins and transporters downregulated genes). Interestingly, both rejection types showed similar pathways modifications.

Focusing on TBBs, the analysis highlighted that 121 out of the total 19,000 genes under study had a significantly different expression (FDR < 0.05, −1.5 < fold change < +1.5) in CR as opposed to NR: 80 were upregulated and 41 were downregulated.

Moreover, 499 DEGs were detected in CR, compared to AR, 114 in CR vs. NR, and 16,643 in CR vs. D. The details of the comparison between populations and upregulated genes are summarized in Table 2. The investigation of the biological role of genes involved in cell metabolism and inflammation was performed using the IPA software after the modification of the fold change values and padj value (FDR, false discovery rate) from <0.05 to <0.01 in NR vs. D.

**Figure 2:**
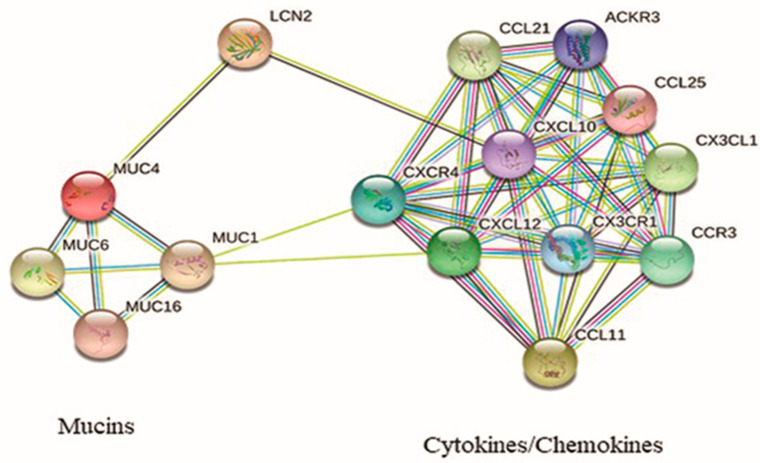
Interaction network of protein encoded by the up-regulated genes in CLAD.

After applying the Benjamin-Hochberg correction for false discovery rate (*p* < 0.05), three significant pathways were found in the comparison of CR vs. AR, five significant pathways in the comparison of AR vs. NR, and one significant pathway observed in the comparison of NR vs. D. We focused our attention on genes involved in metabolic networks that were linked to inflammation and immune response, and specifically those up-regulated in CR compared to AR. Our analysis revealed that the three different networks (immune cell trafficking, cell-to-cell signaling, and interaction–inflammatory disease and inflammatory response) had 33, 26, and 36 DEGs, of which 18, 12, and 18 were up-regulated genes, respectively. Among upregulated genes, seven (CCL11, LCN2, MT-CO2, SCARA3, CX3CL1, CXCL12, and MUC4) were chosen for qRT-PCR validation based on their presence in the identified enriched networks and for their known biological link to the enriched genes (Table 3, Figure 2).

### 3.2. qRT-PCR and Immunohistochemistry

**Figure 3:**
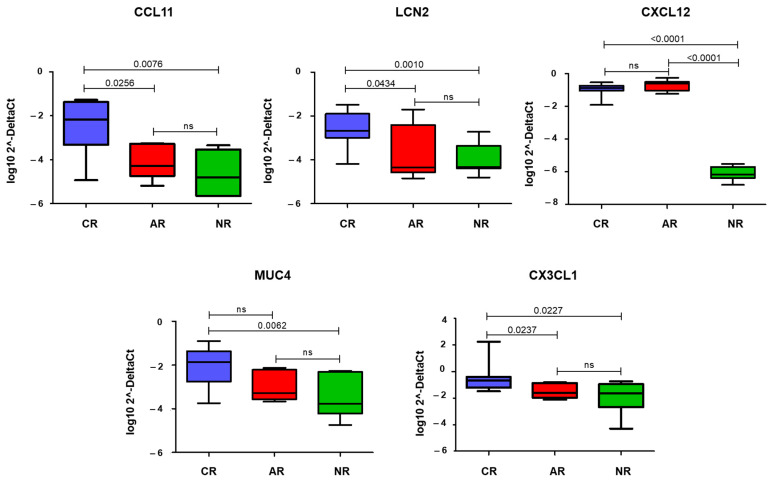
Box-plot showing quantitative Real-Time PCR (qRT-PCR) data of the statistically significant genes in each group of samples. The *y*-axis is a log transformation of the 2^−ΔCt^ values. Horizontal bars show statistical significance (*p* < 0.05) among the comparisons (CR vs. AR, CR vs. NR, and AR vs. NR). CR: chronic rejection; AR: acute rejection; NR: no rejection; ns: not significant.

The comparison between CR and NR samples showed a significant increase in the expression of CCL11, LCN2, CXCL12, MUC4, and CX3CL1 (*p* = 0.008, *p* = 0.001, *p* < 0.0001, *p* = 0.006, and *p* = 0.03, respectively) (Figure 3). When comparing CR and AR, only CCL11, LCN2, and CX3CL1 were found to be significantly different (*p* = 0.03, *p* = 0.04, and *p* = 0.02, respectively) (Figure 3). AR and NR were different only for CXCL12 expression (*p* < 0.0001) (Figure 3).

**Figure 4:**
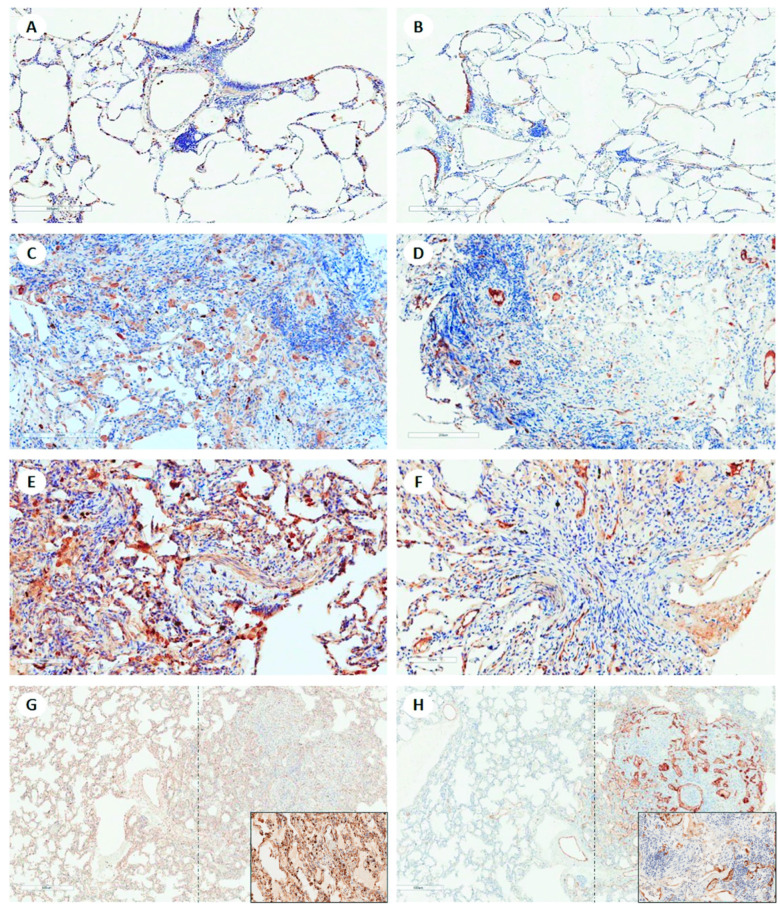
Explicative panel of images of LCN2 and MUC4 immunostaining of lung samples showing different histological features. In donor lung samples, (**A**) LCN2 and (**B**) MUC4 are seen in a few inflammatory cells and in respiratory epithelium, respectively (immunohistochemistry, scale bar: 300 µm). In acute cellular rejection (A3), (**C**) LCN2 is expressed mainly in intra-alveolar macrophages, and (**D**) MUC4 in endothelial cells of a few vessels (immunohistochemistry, scale bar: 200 µm). In obliterative bronchiolitis (ISHLT grading C1) on transbronchial biopsy, (**E**) strong immunostaining of lipocalin 2 is seen in several cell types (epithelial cells of alveolar wall and airways, and inflammatory cells). (**F**) MUC 4 is detected in epithelial and aberrantly in endothelial cells of many vessels (immunohistochemistry, scale bar: 100 µm). In CR (mixed phenotype: the thickening of interstitial spaces with NSIP-like features on the left of the dotted line and features of obliterative bronchiolitis on the right of the dotted line), (**G**, inset) LCN2 is strongly expressed in many inflammatory cells and epithelial cells, and (**H**, inset) MUC 4 is seen in many epithelial and endothelial cells (immunohistochemistry, scale bar: 600 µm).

Among the key markers identified from transcriptomic analysis, LCN2 and MUC4 showed a higher expression in CR than in AR or NR, even if not statistically significant (Figure 4). Interestingly, in CR samples, the staining was aberrantly expressed in several cell types, including epithelial cells (LCN2) and endothelial cells (MUC4) (Figure 4). CXCL12 was equally expressed in AR and CR. CX3CL1 and CCL11 were seldom expressed, mainly in inflammatory cells, without differences among groups.

Morphometrical quantification of LCN2 and MUC4 expression in explanted lungs with noticeably visible CR signs showed a mean (±SD) % of 3.9 ± 3.3 and 3.0 ± 3.0, respectively.

**Figure 5:**
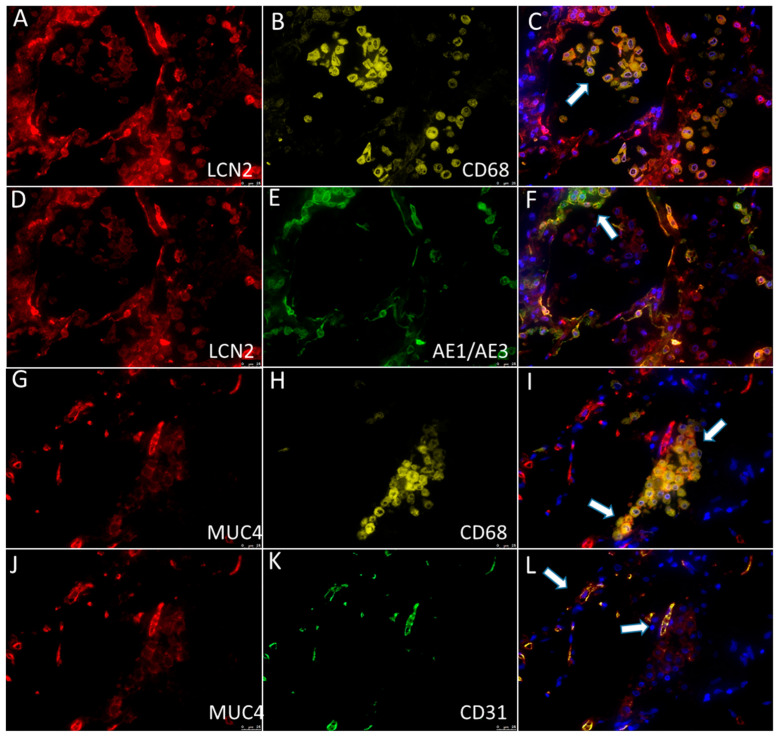
Multiplex immunofluorescence showed the expression of (**A**,**D**; red) LCN2 in (**B**; yellow) CD68+ macrophages (**C**; the overlay image of **A**,**B**; arrow) and (**E**; green) AE1/AE3+ epithelial cells (**F**; the overlay image of **A**,**E**; arrow). Multiplex immunofluorescence showed the expression of (**G**,**J**; red) MUC4 in (**H**; yellow) CD68+ macrophages (**I**; the overlay image of **G**,**H**; arrows) and (**K**; green) CD31+ endothelial cells (**L**; the overlay image of **J**,**K**; arrows).

Co-localization of LCN2 with macrophages and epithelial cells, as well as MUC4 with macrophages and endothelial cells, was noticeably visible in multiplex immunofluorescence (Figure 5).

### 3.3. Finding of Longitudinal Cases

Focusing on patients who developed CR in the study’s follow-up, a sort of expression gradient was found among the different TBBs of each patient, even if not statistically significant due to the low number of cases (data not shown).

## 4. Discussion

The application of molecular analysis, and particularly the use of high throughput technologies, is expected to produce important contributions, including in the LTx field [7,8,9,10]. These analyses first require an appropriate selection of cases avoiding likely biases and a careful analysis of the biological materials to process, as is currently being undertaken in the oncological field. For this reason, in our study we decided to include TBBs with different histological features, including those with true changes of CR in CF patients with CLAD.

In the present study, five genes (LCN2, CCL11, MUC4, CX3CL1, and CXCL12) were found to be up-regulated in CR samples and, notably, the innate immune response was the predominant pathway highlighted by the up-regulated genes. It is noteworthy that two of these genes were also found to be expressed in immunohistochemistry: LCN2 and MUC4.

LCN2 (Lipocalin 2 or NGAL, neutrophil gelatinase associated lipocalin) plays a role in innate immunity by limiting bacterial growth through sequestering iron-containing siderophores. Initially, LCN2 was discovered as a component of the late granules of human neutrophils [23], but is known to be differentially expressed in several human tissues. In lungs, LCN2 is specifically produced by the bronchial epithelium and has been hypothesized as a biomarker in the diagnosis and/or prognosis of various acute and chronic diseases. It is noteworthy that when LCN2 forms a trimeric complex with siderophores and iron, it promotes epithelial-mesenchymal transition (EMT), an important process involved in CR of several solid organ transplantations.

In a recent study, LCN2 was found to be highly expressed in the serum and in the bronchial epithelium and alveolar walls of RAS patients. Moreover, serum LCN2 was proposed as a biomarker for CLAD prediction, being associated with a worse freedom-from-CLAD after LTx [24].

MUC4 (Mucin 4) is a highly glycosylated integral membrane protein, situated on the cell surface. It has been reported to be overexpressed in the lung tissue of patients with idiopathic pulmonary fibrosis. Together with transforming growth factor-β1, MUC4 promotes the remodeling of the alveolar epithelium and EMT [25]. An intriguing finding obtained by immunohistochemistry from our cases was aberrant MUC4 expression also occurring in the endothelial cells of several blood vessels. MUC4 expression has also been observed in the luminal surface of the endothelium of blood vessels where it plays a protective role, providing a non-adhesive surface [26]. Experimental models have shown MUC4 expression in injured endothelial cells, as that occurring in damaged rat cornea [27]. In vitro and in vivo experiments, as well as human data from kidney recipients, have shown that the binding of DSA to endothelial cells profoundly affects the endothelial transcriptome, favoring the expression of several pathways involved in endothelial mesenchymal transition-EnMT [28].

In a model of pancreatic cancer, Kaur et al. [29] demonstrated that LCN2 expression is tightly regulated by MUC4 through the stabilization of the HER2/AKT/NF-κB pathway. In CLAD, these could synergistically work to induce both EMT and EnMT, accumulation of fibrotic tissue, and fibrous remodeling in lung parenchyma and airways [3,30]. In the predictive protein network that we investigated using the STRING database [31] (Figure 2), LCN2 seems to have a central role in connecting two groups of biological molecules, mucins and chemokines, with their respective receptors. It can be speculated that the overexpression of MUC4 observed in CLAD, which in turn promotes LCN2 synthesis and secretion, leads to the recruitment of a pool of chemokines/chemokine receptors (among which would be CCL11, CX3CL1, and CXCL12) already known to be responsible for leukocyte blood extravasation and migration to the inflamed tissue and EMT [32,33]. This would support the hypothesis that LCN2 is a crucial protein in the progression of the LTx from the AR to CLAD disease. Indeed, LCN2 involvement in different types of cancers has led pharmacological research to focus on different mechanisms to inhibit LCN2, both at the gene and protein level [34].

Unlike the result obtained in molecular analysis, CXCL12, CX3CL1, and CCL11 did not show any significant protein over-expression in CR samples. mRNA is usually translated into protein under the assumption that there is some sort of correlation between the level of mRNA and level of protein. However, there may be reasons for this discrepancy, for example a post-transcriptional block. Moreover, proteins can have very different half-lives as the result of varied protein syntheses and degradations, depending on different conditions. Future in-depth studies are needed to investigate the post-transcriptional activity of these proteins, both in vitro and in vivo. Additionally, there could be a problem concerning immunostainings. The antibodies used in this study are not routinely employed in diagnostics, and there is no standardization for defining their positivity. In this regard, further studies are required considering that the absence of a cut-off value may influence the detection rate of positive samples.

Our study had several limitations. First, we performed our experiments with a limited number of patients. However, an important strength of our study was to have collected a large number of TBBs, including the donor-biopsy of most CF recipients taken at the time of the lung transplantation (time 0). Second, our study population was composed only of early and severe CLAD, within the three years post-LTx. This did not allow us to evaluate the effects of any long-term factors, such as infections or different therapeutic regimens. Nevertheless, since the patients were all followed-up in a single center, the therapeutic scheme was always the same and only skilled personnel was used for the clinical management, thus limiting bias. Third, we studied gene expression on RNA extracted from FFPE LTx biopsy specimens. It is known that a large amount of eluted RNA is degraded; however, our samples contained a great amount of RNA fragments with DV200 > 30%, a value sufficient for the RNA-sequencing technology. An additional limitation related to our technology was the need of a validation of the molecular results. Although we validated our data through PCR and immunohistochemistry, we only analyzed the patients of our study population. Nonetheless, the inclusion of a homogeneous study population affected by the same native disease avoided potential biases (e.g., age-related comorbidities and etiology). However, an independent cohort of CF recipients, patients with other native diseases, and a large sample size is mandatory to confirm the value of such a gene expression profile. Another limitation was that routine DSA monitoring and C4d immunostaining, together with a multidisciplinary approach to AMR, were started in our center only towards the end of 2016. As a consequence, we cannot rule out at this stage that patients with AMR may have been under-recognized. Finally, in this study, we included CR patients without distinction of BOS and RAS. Thus, a large case series would permit the identification of gene transcripts specific for these two phenotypes.

## 5. Conclusions

To the best of our knowledge, this is the first omic study using RNA sequencing conducted on TBBs of CF patients. The results merit further examination in a larger number of TBBs coming from multicenter samples of patients with transplants. Such study would also benefit other end-stage diseases. The integration of clinical, radiological, and transcriptomic findings should bridge important gaps in our knowledge, enabling us to accurately verify the role of these pathways in the pathophysiology of CR and possibly discover novel biomarkers for its early diagnosis and treatment.

## Figures and Tables

**Table 1:** Demographic data and clinical characteristics of the study population.

	Study Population (18 Patients)
Age (years) [mean ± SD]	26.1 ± 10.1
Sex (males:females)	6:12
BMI (kg/m^2^)	19.0 ± 3.0
Colonization at the time of transplantation (yes:no)	14:4
FEV1 (L)	1.0 ± 0.3
Donor age (years) [mean ± SD]	32.0 ± 12.8
Donor sex (males:females)	9:9
Mean ischemic time (min) [mean ± SD]	326.7 ± 89.9
CMV status [N (%)]	
D^+/−^ R^+^	14 (78%)
D^+^ R^−^	0 (0%)
D^−^ R^−^	4 (22%)
CLAD [N (%)]	12 (67%)
Status (dead:alive)	6:12
Survival (years) [mean ± SD]	6.4 ± 3.2

SD, standard deviation; BMI, body mass index; FEV1, forced expiratory volume in the 1st second; CMV, cytomegalovirus; D, donor; R, recipient; CLAD, chronic lung allograft dysfunction.

**Table 2:** Differential gene expression analysis [adjusted *p*-value (FDR) < 0.05: −1.5 < Fold Change < + 1.5].

Comparison	Total DEGs	Down-Regulated Genes	Up-Regulated Genes
D vs. NR	15,444	11,680	3764
D vs. AR	18,452	13,808	4644
D vs. CR	16,643	11,198	5445
CR vs. NR	114	37	77
CR vs. AR	499	275	224
AR vs. NR	94	28	66

DEG, differentially expressed genes; D, donor lung biopsies; NR, no rejection biopsies; AR, acute cellular rejection biopsies; CR, chronic rejection biopsies.

**Table 3:** Transbronchial biopsies with chronic rejection vs. acute rejection: crucial genes related to inflammatory and regulatory signatures.

Gene Symbol	Gene Description	Relative Expression		
		Log2 Fold Change	*p*-Value	padj
CCL11	C-C Motif Chemokine Ligand 11	3.2	8.6 × 10^−8^	4.8 × 10^−5^
LCN2	Lipocalin 2	2.7	1.0 × 10^−4^	8.0 × 10^−3^
MT-CO2	Mitochondrially Encoded Cytochrome C Oxidase II	2.8	7.3 × 10^−4^	2.9 × 10^−2^
SCARA-3	Scavenger Receptor Class A Member 3	1.3	4.6 × 10^−6^	9.2 × 10^−4^
CX3CL1	C-X3-C Motif Chemokine Ligand 1	1.5	8.0 × 10^−5^	6.7 × 10^−3^
CXCL12	C-X-C Motif Chemokine Ligand 12	1.2	1.1 ×10^−5^	1.6 × 10^−3^
MUC4	Mucin 4	2.7	7.4 × 10^−6^	1.3 × 10^−3^

## Data Availability

Raw data are available in SRA.

## Abbreviation List

ACR: acute cellular rejection; AMR, antibody-mediated rejection; BAL, bronchoalveolar lavage; BMI, body mass index; CF, cystic fibrosis; CLAD, chronic lung allograft dysfunction; CMV, cytomegalovirus; CR, chronic rejection; D, donor; DEG, differentially expressed genes; EMT, epithelial-mesenchymal transition; FC, fold change; FDR, false discovery rate; FEV1, forced expiratory volume in 1 s; FFPE, formalin-fixed paraffin-embedded; IHC, immunohistochemistry; ISHLT, International Society of Heart and Lung Transplantation; LCN2, lipocalin 2; LTx, lung transplantation; MUC4, mucin 4; NR, no rejection; NSIP, non-specific interstitial pneumonia; PPFE, pleuro-parenchymal fibroelastosis; R, recipient; RAS, restrictive allograft dysfunction; RT-PCR, real-time polymerase chain reaction; SD, standard deviation; TBB, transbronchial biopsies.
